# Membrane-camouflaged biomimetic nanoparticles as potential immunomodulatory solutions for sepsis: An overview

**DOI:** 10.3389/fbioe.2023.1111963

**Published:** 2023-03-10

**Authors:** Yanbei Wang, Liping Liu, Xinchuan Zheng, Xin Liu

**Affiliations:** ^1^ School of Culture and Tourism, Chongqing City Management College, Chongqing, China; ^2^ Chongqing Institute of Green and Intelligent Technology, Chinese Academy of Sciences, Chongqing, China; ^3^ Medical Research Center, Southwest Hospital, Army Military Medical University, Chongqing, China

**Keywords:** biomimetic nanoparticles, membrane-coating, sepsis, infection, inflammation, immunosuppression, nanotherapeutics

## Abstract

Sepsis is a life-threatening organ dysfunction due to dysregulated host responses induced by infection. The presence of immune disturbance is key to the onset and development of sepsis but has remarkably limited therapeutic options. Advances in biomedical nanotechnology have provided innovative approaches to rebalancing the host immunity. In particular, the technique of membrane-coating has demonstrated remarkable improvements to therapeutic nanoparticles (NPs) in terms of tolerance and stability while also improving their biomimetic performance for immunomodulatory purposes. This development has led to the emergence of using cell-membrane-based biomimetic NPs in treating sepsis-associated immunologic derangements. In this minireview, we present an overview of the recent advances in membrane-camouflaged biomimetic NPs, highlighting their multifaceted immunomodulatory effects in sepsis such as anti-infection, vaccination, inflammation control, reversing of immunosuppression, and targeted delivery of immunomodulatory agents.

## Introduction

Sepsis represents a severe and heterogeneous clinical syndrome that is described as life threatening organ dysfunction resulting from a dysregulated host response to infection (Singer et al., 2016). As a global health priority recognized by WHO, sepsis affects tens of millions of people worldwide and represents almost 20% of deaths on Earth each year (Reinhart et al., 2017; Rudd et al., 2020). Immunopathy is central to the pathogenesis of sepsis, which is characterized by a profound immune imbalance involving both hyperinflammation and immunoparalysis that disturb the homeostasis of the host and promote life-threatening organ injury (van der Poll et al., 2017; van der Poll et al., 2021). Specifically, sepsis occurs when an abundance of microbial cell-wall components, namely, pathogen-associated molecular patterns (PAMPs), along with cellular injury and the secretion of danger associated molecular patterns (DAMPs)—bind to pattern recognition receptors to initiate a network of inflammatory responses that promote tissue injury and organ dysfunction. Meanwhile, sepsis also induces systemic and sustained immunosuppression, as shown by tolerant reprogramming of innate immune cells, expansion of suppressive myeloid cells, and overwhelming apoptosis and exhaustion in T lymphocytes, resulting in increased susceptibility to secondary infection (Wang et al., 2022). These advances in our understanding of how sepsis occurs and develops have prompted translational investigations into the efficacy of immunomodulatory strategies. Unfortunately, few attempts have been considered successful despite decades of effort. Treating sepsis is still with remarkably limited therapeutic options (van der Poll et al., 2017). Therefore, innovative strategies are in increasing demand to achieve effective immunomodulation, which may help to improve the clinical outcome of sepsis.

Given the inherent challenges that impede the efficacy of conventional therapeutic agents such as poor bioavailability and lack of specific targeting, the emerging nanoparticle (NP)-based therapeutics have revolutionized the biomedical field, providing alternative approaches that may dramatically overcome the deficiencies of traditional therapies. Specifically, the controllable physicochemical features of NPs, including size, shape, and surface properties, lead to highly increased stability and bioavailability (Guo et al., 2021; Pant et al., 2021). Further, the surfaces of NPs can be diversely functionalized, which can greatly improve their capacity for targeted delivery to specific cells or tissues. These advantages have thus prompted research into develop promising immunomodulatory nanosystems that facilitate the adjunctive therapy of sepsis and other immunological disorders (Feng et al., 2019; Lasola et al., 2020). Non-etheless, naked NPs may be rapidly internalized by phagocytes (e.g., monocytes and macrophages) before performing their intended function. Their potential contact-toxic effect is another concern for their broad application. In this case, the development of biomimetic NPs may provide an alternative solution. Biomimetic NPs are artificially manufactured hybrids with both nano and natural materials (Meyer et al., 2015; Thorp et al., 2020). A typical biomimetic NP is made up of a definite core nanostructure that mimics the size and shape of cells or microbes, along with a camouflaging surface structure composed of biological membranes or membrane-anchored components (Meyer et al., 2015). Importantly, biomimetic NP are increasingly used for immunomodulatory purposes in treating sepsis, including inflammation targeting (Jin et al., 2018), anti-infection (Zhao et al., 2021), and immune cell activation (Lasola et al., 2020). In this regard, this minireview discusses the basic features of biomimetic NPs and their recent advances in developing potential remedies for sepsis treatment while highlighting the biomimicry properties underlying their immunomodulatory effect.

## Nanomaterials for the management of sepsis

The successful management of sepsis relies heavily on early diagnosis and effective intervention. Nanotechnology has become increasingly recognized to meet these demands due to the inherent properties of nanomaterials that enable 1) imaging of infection, 2) capture and clearance of pathogens, PAMPs and DAMPs, 3) targeted delivery of therapeutic agents for antimicrobial, anti-inflammatory, anti-oxidant and immunomodulatory purposes (Pant et al., 2021; Papafilippou et al., 2021). First, many classes of NPs are used as core materials in sepsis management, including polymers, liposomes, metals and inorganic NPs. Metal NPs are preferentially utilized for the diagnosis of infection due to their unique optic features (Pant et al., 2021). Polymeric NPs and liposomes are more frequently employed to encapsulate therapeutic agents to increase bioavailability, tolerance and targeting (Papafilippou et al., 2021). For example, FDA has approved the use of liposomal formulations containing anti-fungal amphotericin B to treat fungal infection, an increasingly frequent cause of sepsis (Stone et al., 2016). Second, the sizes of these NPs are diversified based on the context of application in treating sepsis. Specifically, smaller particles (5–20 nm) are preferentially used for diagnosis while larger particles (100–200 nm) are mostly employed as targeted drug carriers or for clearing purposes. Third, functionalization with chemical or biological moieties is another feature that decide the use of NPs in sepsis. Indeed, functionalized NPs are advantaged to naked NPs by demonstrating decreased toxicity, increased retention and targeted delivery. These advantages are important for enhancing the efficacy and specificity of cargo antibiotics or anti-inflammatory agents. In particular, there is growing interest to develop biomembrane-camouflaged NPs for treating sepsis (Ismail et al., 2022). These particles not only preserve the required advantages of synthetic nanomaterials by also inherit the inherent beneficial features of natural biological surfaces, such as decreased immunogenicity and toxicity, increased blood retention, and controllable targeting (Meyer et al., 2015).

## A general description of membrane-coated immunomodulatory biomimetic nanoparticles

**FIGURE 1:**
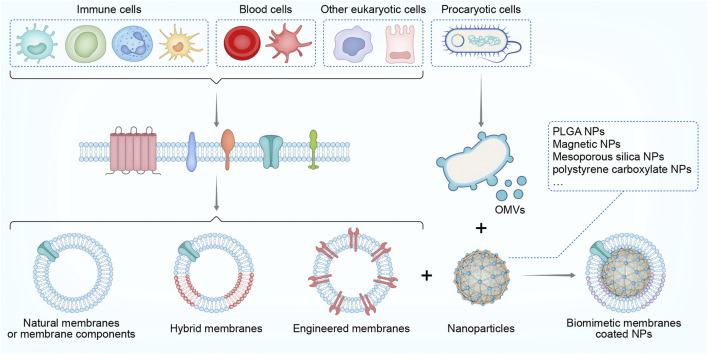
Scheme of cell membrane-coated NPs used in sepsis treatment: The origin of biological membranes; the assembly of membranes with core NPs to form biomimetic NPs. NP, nanoparticles, PLGA, poly (lactic-co-glycolic acid) OMVs, outer membrane vesicles.

Nanoparticles become biomimetic when coated or camouflaged with biological membranes that form core-shell structures, leading to combinatory activity featuring both the versatility of nanomaterials and the functionality and biocompatibility of the biomembrane system (Jin et al., 2018; Sushnitha et al., 2020). Hence, coating with a biomimetic membrane may greatly improve the stability, retention, and combability of naked NPs. Meanwhile, the selective utilization of membrane-targeting dependent mechanisms preserves a variety of bioactivities such as recognition, binding, delivery, or cellular modulation while avoiding potentially unfavorable effects due to the absence of cellular contents (Thorp et al., 2020; Oroojalian et al., 2021). Generally, biomimetic NPs are manufactured based on the following elements: 1) a core nanostructure of synthetic polymers or liposomes that are stable, inert, or cell-mimetic (e.g., size and shape); 2) a coated biomembrane, membrane-like structure, or membrane components (e.g., proteins and polysaccharides); and iii) an assembly strategy for loading and coating separated biological membranes onto nanomaterials. The representative NPs coated with biological membranes or membrane components currently applied in sepsis therapy are summarized in Figure 1.

### Types of biomimetic shells/membranes

The biomimetic shells are critical for NPs to behave like real cells or for a designated function at the interface between biological cells or molecules (Fang et al., 2018; Sushnitha et al., 2020). The concept of shell-coating originated from NP PEGylation, which is the process of creating a hydration layer with polyethylene glycol (PEG) polymers to increase NP stability and reduce toxicity (Fang et al., 2018). Subsequently, NPs have been decorated with a variety of targeting ligands—ranging from antibodies and antigens to nucleic acid and small molecular compounds—for the extended purposes of cell recognition, targeted delivery, and modulation of cell functions (Sushnitha et al., 2020). Furthermore, in one study, an entire membrane was separated and assembled onto NPs, which thus comprehensively recapitulated the properties and functions of cellular surfaces (Sushnitha et al., 2020). Here, we briefly introduce biomimetic membranes that are preferentially used with immunomodulatory purposes. Most of biomimetic membranes are derived from eukaryotic cells, although a few are isolated from prokaryotic organisms (Meyer et al., 2015). Specifically, both naïve cell membranes and those derived from activated or genetically engineered cells can be utilized to coat NPs, whether to simply evade premature clearance or to improve the targeting capabilities to specific sites or objects (Sushnitha et al., 2020). Immune cells such as macrophages, T cells, and neutrophils are among the most widely used eukaryotic source cells for obtaining membranes camouflaged on NPs (Oroojalian et al., 2021). These immune-cell biomimetic NPs may possess the advantageous surface functions of immune cells, such as selective targeting, immune boosting, and intercellular communication; therefore, they are widely utilized for reversing immune disorders in tumors, autoimmune diseases, cardiovascular complications, and sepsis (Jahromi et al., 2021). Blood cells are another typical source of eukaryotic cell membranes. In particular, NPs have been coated with wild-type membranes from red blood cells (RBCs), platelets, and leukocytes (Parodi et al., 2013; Feng et al., 2020; Jiang et al., 2020), as well as hybrid membranes of blood cells and other tissue cells (Huang et al., 2022), aiming for prolonged retention, increased tolerance and targeted delivery to pathogens or tissues for immunotherapy. In addition to eukaryotic cell membranes, biomimetic NPs inspired by prokaryotic membrane structures such as bacterial outer membrane vesicles (OMVs) have also been designed and manufactured, attempting to enhance antibacterial vaccination (Anwar et al., 2021), improve anti-infectious therapies (Gao et al., 2022), and augment anti-inflammatory activities (Gao et al., 2022) in the host. Sometimes, isolated or genetically obtained membrane components such as surfactants (Wang et al., 2020), exosomes (Li et al., 2022), and polysaccharides (Zandi et al., 2020) may also be obtained for specific targeting or biomimetic modulatory purposes.

### Core particles for membrane coating

Candidate NPs are also important as the inner core structure for fully realizing the biomimetic functions by coated with biological membranes, although their preparation is not distinct compared to that of naked NPs (Liu et al., 2019). Inert and biocompatible NPs are often given priority for membrane encapsulation, which mainly includes organic polymers and liposomes as well as inorganic materials like metal NPs and iron oxide (Liu et al., 2019). For example, poly (lactic-co-glycolic acid) (PLGA) NPs, gelatin and polystyrene/polypropylene (PS/PP) NPs are extensively used as the inner cores for the coating of immune cell membranes and blood cell membranes (Oroojalian et al., 2021). Actually, some of these organic polymers and their applications have been approved by the FDA due to their excellent biocompatibility and the capacity as drug carriers (Liu et al., 2019). Meanwhile, inorganic materials (e.g., gold, selisuperparamagnetic iron oxide) have also been introduced owing to their unique photothermal and photodynamic abilities, which may assist or even strengthen the immunomodulatory effects (Liu et al., 2019). In addition to types, the sizes of biomimetic NPs also affect are adjustable in context dependent manners (Narain et al., 2017). Smaller particles (50–200 nm) employs surface area and are able to reach targeted cells more easily used for vaccination, antigen presentation and targeted delivery. Larger particles (>300 nm) may better recapitulate the structure of cells to capture pathogens or facilitate immunoregulation by facilitating direct contact with immune cells. In addition, the shapes of biomimetic NPs are not diversified as synthetic NPs. Actually, most of the core materials for biomimetic NPs are round shape so that they are apparently better encapsulated with cell membranes and perform like real cells.

### Assembly strategies

Obtaining a perfect membrane coating of the core materials is another key factor that affects the functionality of biomimetic NPs. A recent study indicated that up to 90% of examined biomimetic NPs were incompletely coated, which may dramatically affect the efficiency of their endocytic entry (Liu et al., 2021). Recent review articles have well summarized the principal methods of preparing biological membranes and assembling them onto NP cores (Fang et al., 2018; Choi et al., 2020). Herein, we only briefly discuss the key points of coating the NP core with biological membranes after membrane preparation, as realized by cell rupture and extraction. Inspired by the synthesis of liposomes, mechanical extrusion and sonication are amongst the most widely employed methods for membrane coating (Choi et al., 2020). Although these methods are simple and controllable, the infliction of physical forces may either affect the integrity of the coating membranes and core materials or break the uniformity of the synthesized particles (Liu et al., 2021). Other preparation methods, such as microfluidic electroporation (Rao et al., 2017), intracellular coating, and cell membrane-templated polymerization, have also been developed to avoid these deficiencies (Choi et al., 2020). However, further verifying experiments are needed to confirm their effectiveness.

### Immunomodulatory properties

Immunomodulation has become an emerging modality for the application of membrane-coated NPs, where the central strategy is to mimic natural immune responses while minimizing unwanted effects such as premature clearance and immunotoxicity (Sushnitha et al., 2020). Multifaceted purposes have been achieved with the coating of biological membranes or membrane-derived agents, such as activating immune cells, remodeling the immune microenvironment, intensifying vaccination, alleviating inflammation, and facilitating targeted delivery (Sushnitha et al., 2020). Meanwhile, coating with either immune cells or blood cells may also make the encapsulated particles more tolerant and retentive. One example of immunomodulation is realized by immune cell membranes anchored by stimulatory molecules. For example, PEGylated bilirubin NPs coated with M1-macrophage membranes or mesoporous silica NPs coated with dendritic cell membranes favor cytotoxic T cell generation *via* mimicking the interaction of co-stimulatory molecules between antigen presenting cells and lymphocytes (Hu et al., 2020; Li et al., 2021). Furthermore, NPs have been exploited to activate innate and adaptive immunity when coated with bacterial membranes carrying PAMPs. In one study, an injection of gold NPs encapsulated with OMVs from *Escherichia coli* rapidly activated dendritic cells in the lymph nodes of mice while selectively promoting Th1 and Th17 immune responses (Gao et al., 2015). Immunomodulatory strategies can be also achieved by remodeling the immune microenvironments *via* fabricating biomimetic NPs with specific immunological membranes or membrane moieties, which can repolarize macrophages (Yue et al., 2021), promote antigen exposure by inducing lytic cell death (Chen et al., 2022), or release immunostimulants into the microenvironment (Zhuang et al., 2019). Another prominent approach is mainly realized by the membrane-dependent targeted delivery of anti-infectious and anti-inflammatory agents to specific sites (Yang et al., 2019; Sushnitha et al., 2020; Zhang et al., 2021). For example, RBC and platelet membranes are preferably employed to coat antimicrobial NPs or inert NPs carrying antibiotics, which are delivered to the targeted infectious or inflamed site based on the binding activity of RBCs or platelets towards bacteria.

## Immunomodulation by membrane coated NP in sepsis

**FIGURE 2:**
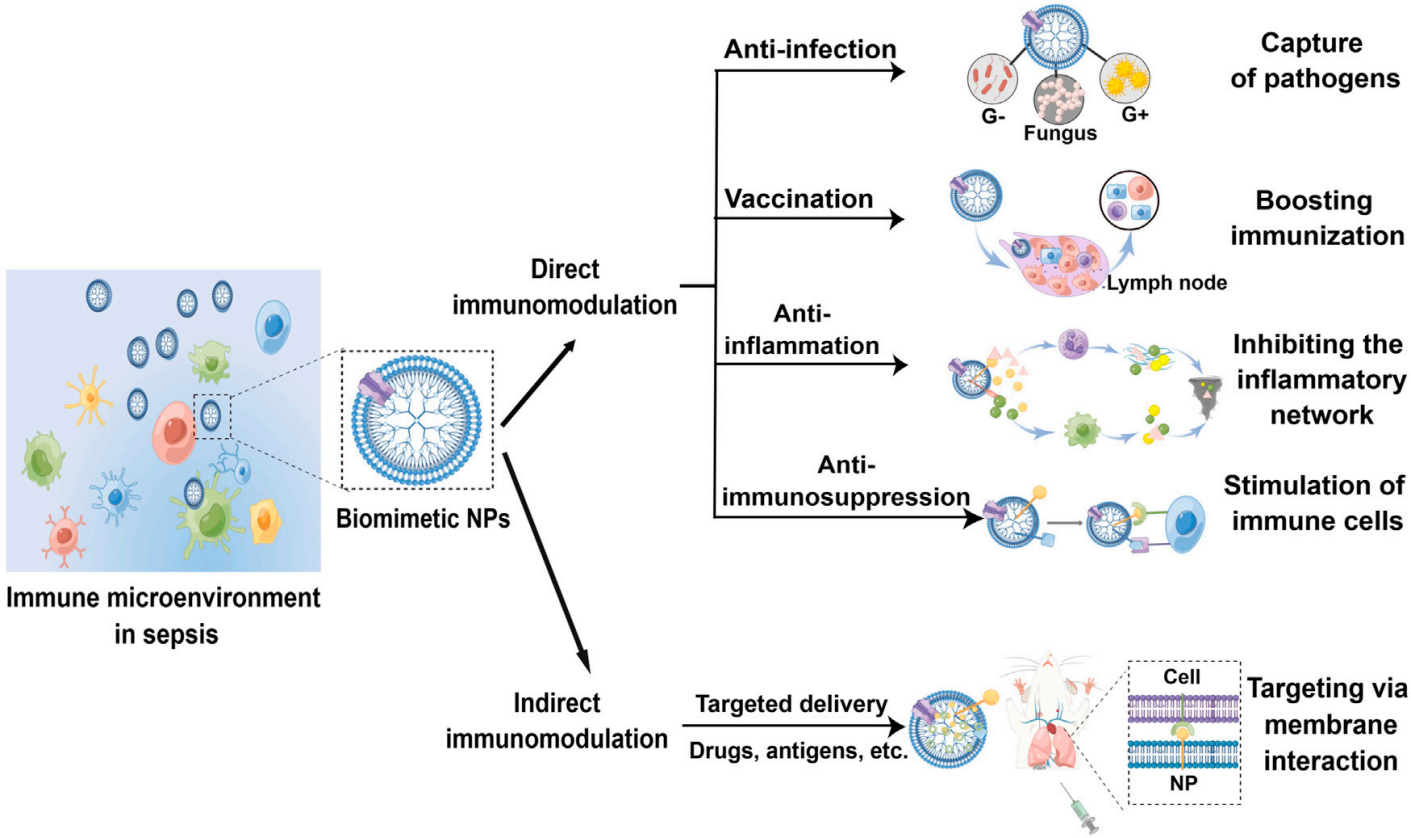
Schematic of the direct and indirect immunomodulatory effect by biomimetic NPs in sepsis treatment (By Figdraw).

Sepsis is characterized by a dysregulated immunity that originates from infection, whose development involves the production of PAMPs, toxins, DAMPs, and inflammatory mediators that harm the host *via* a concurrent course comprising both excessive inflammation and immunosuppression (van der Poll et al., 2021). In this regard, immunomodulatory effects may be realized directly by biomimetic NPs to reverse these abnormities. Furthermore, other indirect mechanisms are also employed, such as the targeted delivery of immunomodulatory agents or augmenting their bioactivity. Table 1 and Figure 2 summarize the immunomodulatory effects of biomimetic NPs in sepsis treatment. In Table 1, the features of NPs and models regarding the purposes of immunomodulation has also been describes.

**TABLE 1:** Immunomodulatory biomimetic NPs in sepsis.

Immunomodulatory purposes	Types of membranes	Type of NPs	Size (nm)	Single dose	Experimental models	Mechanisms of action	Ref
Anti-infection	RBC	Gelatin	152	2 mg/kg	MRSA infected mice	Monitoring infection and antibacterial effect	Lin et al. (2019)
	PLT&PMN	Null	356	1 × 10^11^ pfu/rat	*E. coli* infected rat	Phage delivery	Jin et al. (2021)
	MΦ	PLGA	102	200 mg/kg	LPS & *E. coli* injected mice	Absorbing LPS and cytokine	Thamphiwatana et al. (2017)
Vaccination	OMV	AuNP	41.9	2.5 μg gold/mice	Vaccinated mice	Membrane vaccination	Gao et al. (2015)
	PMN	PLGA	160	100 μg NP/mice	Mice with pneumonia	Capture and delivery of OVA	Zhou et al. (2022)
Anti-inflammation	MΦ	PLGA	155.2	15 mg/kg	Poly I:C injected mice coronavirus infected mice	Neutralizing cytokines	Jiang et al. (2022)
	MΦ	PLGA	102	20 μg/mice		Absorbing cytokines and antiviral effect	Tan et al. (2021)
	MΦ	Liposome	94	1.8mM/mice	LPS injected mice	Modulation of gene expression	Molinaro et al. (2019)
Anti-immunosuppression	MΦ	ZIF/MOF	160	10 mg/kg	Immunocompromised mice infected by MDRSA & *E. coli* MRSA systemic infected mice	Boosting the generation of the immunostimulatory LL37	Cao et al. (2022)
	PLT	PLGA	120	100 μg/mice		Preserving the function of immune cells	Kim et al. (2021)
Targeted delivery	RBC	PLGA	226.47	100 μg/kg	CLP in mice	Delivery of bFGF	Li et al. (2022b)
	Engineered cell	PLGA	175	20 μg/mice	LPS injected mice	Targeted delivery of dexamethasone	Park et al. (2021)

RBC, red blood cell; MRSA, methicillin-resistant *Staphylococcus aureus*; PLT, platelet; PMN, polymorphonuclear leukocyte; MΦ, macrophage; PLGA, poly (lactic-co-glycolic acid); LPS, lipopolysaccharide; OMV, bacterial outer membrane vesicle; AuNPs, gold nanoparticles; PS-COOH, polystyrene carboxylate; OVA, ovalbumin; poly I:C, polyinosinic: polycytidylic acid; MDRSA, multiple drug resistant *Staphylococcus aureus*; bFGF, basic fibroblast growth factor.

In general, RBC and macrophage membranes are preferable sources of coating membranes while PLGA NPs are commonly chosen as the core material. In addition, the particle sizes usually exceed 100 nm, making them retentional and readily available for any immunomodulatory purpose in sepsis treatment. In addition, the doses are decided by the cargo the NPs carry and by the definite roles of immunomodulation. For example, immunization requires far less amounts of NPs than anti-infectious or anti-inflammatory purpose. The use of laboratory sepsis models is another key aspect. Sepsis models can be simply classified as injection models and surgical models due to their methodology differences. In the injection models, pathogens or pathogen products [lipopolysaccharide (LPS), *etc.*] are locally or systemically administrated to induce sepsis. In the surgical model, the host barriers are disrupted by surgical operations, allowing a natural course of endogenous infection and sepsis (Wang et al., 2022). The cecal ligation and puncture (CLP) model is the most widely used surgical model in which sepsis is recapitulated by the surgery of cecal ligation and needle puncture that induce abdominal infection. The CLP model replicates a complete course of sepsis and is particularly suitable for evaluating the clinal relevance of immunomodulatory properties of biomimetic NPs (Wang et al., 2022). However, the inconsistency of outcomes may greatly hinder its wide use (Wang et al., 2022). In contrast, bacteria or LPS injection is relatively simpler and more stable. They can also be used to evaluate the specific purposes of targeted therapy. For example, the efficacy against methicillin-resistant *Staphylococcus aureus* (MRSA) sepsis can be examined by directly injection of MRSA in model animals. Therefore, these models are more preferentially used to preliminarily examine the immunomodulatory efficacy of biomimetic NPs in sepsis treatment.

### Anti-infection

#### Direct antimicrobial activity

**FIGURE 3:**
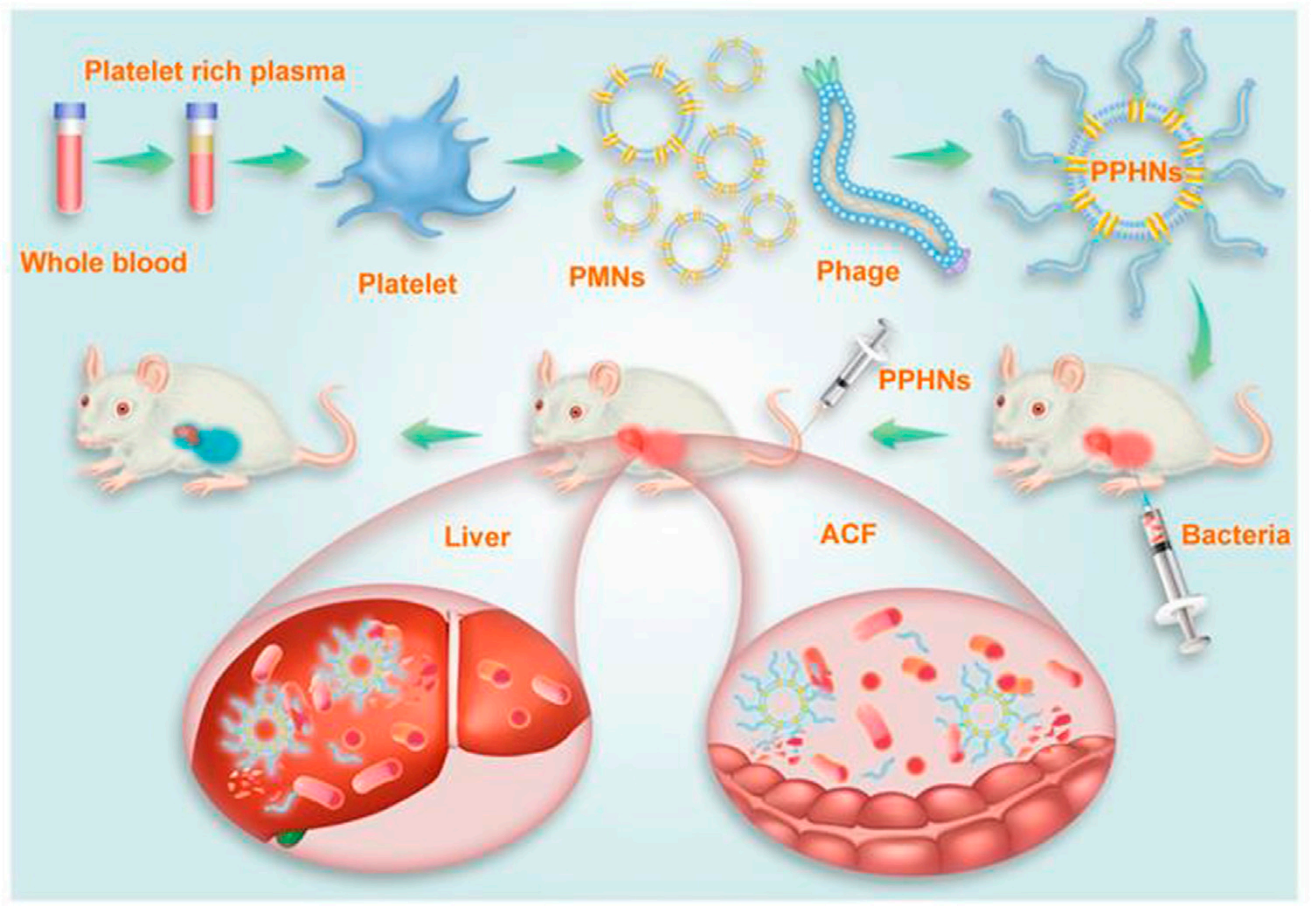
Schematic illustration of PPHNs’ preparation and prolonged anti-bacterial action. The fabricated biomimetic phage-platelet hybrid nanoparticle (PPHN) was designed through the physical binding of the BCP1-BGL phage to the platelet membrane nanoparticles derived *via* a repeated freeze-thaw. The resulting PPHNs with each spherical membranous nanoparticle harboring approximately 12 rod-shaped phage particles stably bound to its surface, were superior to the BCP1-BGL phages that displayed significantly prolonged anti-bacterial action *in vivo* against *Escherichia coli* infection, exhibited further extended blood retention time and optimal anti-bacterial performance. The hydrodynamic size of PPHNs were averaged at 368 nm in distill water and the injection dose of PPHNs was 1 × 10^11^ pfu of phages in the *E. coli* infection model. Reproduced with permission from Jin et al.(2021). Copyright^©^ 2021. The authors. Ivyspring International Publisher.

It is known that use of NPs can directly trap and kill pathogens, and coating with biological membranes or membrane components may strengthen their intrinsic contact-killing capacities against viral and bacterial pathogens most likely through increasing blood retention, avoiding immune clearance, facilitating targeted delivery, or otherwise synergizing to combat infections (Yang et al., 2019). For example, a bacteria-responsive biomimetic selenium nanosystem was developed by Lin et al. (2019) by coating bacteria-responsive Ru-selenium-gelatin NPs with RBC membranes. This system could effectively deliver Ru-complex-modified nano selenium to the infection sites while avoiding premature clearance by the immune system. Furthermore, Jin et al. (2021) designed and fabricated biomimetic phage-platelet hybrid nanoparticle (PPHN) with antibacterial purposes. By coupling platelet membrane NPs with a genetically engineered phage display system, PPHN was demonstrated with prolonged blood retention and better anti-bacterial activity against *Escherichia coli* infection (Figure 3). On the other hand, biomimetic NPs are also utilized to overcome the disadvantages of conventional antimicrobial therapeutics, such as early degradation, drug resistance and poor targeting (Cano et al., 2020; Chen et al., 2021; Huang et al., 2021). In a recent study, vitamin lipid NPs that carry antimicrobial peptide and cathepsin B (AMP-CatB) mRNA were transfected into macrophages. The NP-containing macrophages were then adopted transferred to eliminate multidrug-resistant *Staphylococcus aureus* and *E. coli* in septic mice (Hou et al., 2020). Additionally, Jabalera et al. (2021) evaluated the antimicrobial efficacy of biomimetic magnetic NPs functionalized with the antimicrobial peptide AS-48. They found that the nanoformulation displayed robust bactericidal activity with reduced dosage and side effects. Cell-membrane-based biomimetic NPs are also used for COVID-19 treatment, aiming to provide nanotechnology tools that inactivate SARS-CoV-2 (Weiss et al., 2020) or increase the therapeutic benefits of repurposed antiviral molecules *via* their distinct advantages over free drugs, such as the increased targeting and bioavailability (Pereira-Silva et al., 2021).

#### Absorption of pathogens, PAMPs and other toxins

The second approach to the anti-infection activity of biomimetic NPs is *via* their capacity to capture and adsorb microbes, PAMPs, and other toxins. Given that pathogens are recognized by host cells *via* the recognition receptors that specifically bind to free PAMPs or conjugated PAMP on microbes, NPs are also coated with RBC, macrophage, or platelet membranes, or modified by pathogen-recognizing molecules, to adsorb pathogens and/or PAMPs. For instance, a recent study reported the conjugation a peptide on superparamagnetic iron oxide nanoparticles (SPIONs) that mimics the binding motif of a human scavenger receptor GP-340c (Friedrich et al., 2022). The biomimetic SPIONs were highly efficient in capturing a broad range of bacteria and bacterial products. Importantly, the bound pathogens could be disassociated for culture and identification, which also highlights a potential application for diagnostic purposes in sepsis. Other studies demonstrated the direct coating of macrophage (Thamphiwatana et al., 2017) or neutrophil (Wu et al., 2020) membranes onto various NP cores to absorb endotoxins and proinflammatory cytokines for treating sepsis. In addition to internal use, biomimetic NPs have also been used as novel adsorbents in extracorporeal blood purification. For example, both magnetic nanobeads coated with engineered human opsonin (Kang et al., 2014) and heparin-conjugated polyethylene beads (Seffer et al., 2020) were assembled to capture pathogens and PAMPs for the treatment of bacterial infection and sepsis. It is well known that bacteria produce and release toxins that injure the host. Therefore, biomimetic NPs are also increasingly applied for toxin removal in sepsis. RBC membranes are among the most extensively used biomimetic membranes for coating NPs and nanosponges. These biomimetic NPs are more stable and less toxic while preserve the ability to absorb bacterial proteins and toxins and neutralize their toxicity, thereby conferring protection against toxin-induced lethality in sepsis models (Chen et al., 2019; Koo et al., 2019; Ben-Akiva et al., 2020; Zou et al., 2022).

#### Vaccination

Effective vaccination strategies are indispensable for refractory infectious diseases prior to sepsis, and using biomimetic nanotechnology may greatly increase vaccine potency (Zhou et al., 2020). Both microbial structures and host-derived components have been considered candidate biomimetic adjuvants that can integrate with antigens to boost the efficacy of vaccination (Zhou et al., 2020). Microbial biomimetic vaccines, also referred to as pathogen-like particles (PLPs), have shown great promise as vaccine delivery platforms (Rosenthal et al., 2014). Rather than the coating of a complete bacterial membrane, PLPs are often produced by inserting antigens inside viral capsids or bacterial OTMs that mimic immunostimulation by viruses or bacteria (Rosenthal et al., 2014). In particular, bacterial OTMs are inherently immunostimulatory and are thus widely utilized as biomimetic membranes for vaccination (Krishnan et al., 2022). For example, gold NPs can be wrapped with *Escherichia coli* OTMs to obtain increased stability and capacity and rapidly promote the maturation of dendritic cells in the lymph nodes of vaccinated mice (Gao et al., 2015). Further, the immunostimulatory molecules expressed on OTMs are also directly used to decorate core particles to produce artificial bacterial biomimetic NPs. Siefert et al. (2016) reported the modification of PLGA NPs with bacterial monophosphoryl lipid A and unmethylated CpG-rich oligodeoxynucleotides, which assisted the model antigen ovalbumin in enhancing the T helper 1 (Th1)-skewed cellular and antibody-mediated responses. Many bacterial toxins are pathogenic and targeted for toxoid vaccination in sepsis, which has promoted the development of nanotoxoid vaccines (Hu and Zhang, 2014). Rather than direct vaccination with purified or recombinant toxins, nanotoxoid vaccines immunize the host by the particle-dependent capture of bacterial toxins that mounts an anti-toxin immune response (Zhou et al., 2022). Hence, the coating of blood cell (RBCs or platelets) membranes may not only stabilize NP cores but also readily enhance their ability to reach the targeted pathogens (Hu and Zhang, 2014). In addition to bacteria-derived membranes or membrane components, NPs can also be coated with mammalian or human-associated membranes or other biological components that serve as adjuvants to increase immunization. In a recent study, RBC-membrane-coated polystyrene carboxylate NPs were demonstrated to couple with the model antigen ovalbumin for a targeted delivery to the spleen, where they robustly promoted antibody production and the memory T cell response (Ukidve et al., 2020). Viral infection and viral sepsis have been increasingly recognized since the outbreak of the pandemic COVID-19. Wu’s group reported the coating of pulmonary surfactants onto liposomes carrying the activation of stimulator of interferon genes (STING) agonists that recapitulated the anti-viral immune response post infection. The pulmonary surfactant coating acted as a mucosal adjuvant that resembled a tolerant surface to increase the penetration of the biomimetic NP vaccine (Wang et al., 2020).

#### Anti-inflammation

Sepsis is initially known by a typical feature of exaggerated inflammation due to an initially hyperactivated immune response. Therefore, inhibitory coated NPs have been developed, and their anti-inflammatory efficacy has been evaluated in various sepsis models (Jin et al., 2018). Membranes from innate immune cells such as macrophages and neutrophils are more frequently used than other blood cells to camouflage core NPs due to their preferential targeting of the inflamed environment and robust anti-inflammatory abilities *via* binding and neutralizing proinflammatory mediators (Molinaro et al., 2019; Pereira-Silva et al., 2021). Chen’s group reported the fusing of a macrophage membrane onto a PLGA nanocore to construct biomimetic NPs, which neutralized proinflammatory cytokines *via* binding to membrane receptors. However, these particles did not activate intracellular proinflammatory signaling pathways that may amplify inflammation due to their lack of cellular content (Jiang et al., 2022). Likewise, Du et al. (2022) invented decoy nanozymes comprising mesoporous silica NP cores loaded with a nanocatalyst and a photosensitizer, followed by further coating with macrophage membranes to create a macrophage-like decoy for inflammatory cytokine neutralization by the core materials. Additionally, macrophage- or neutrophil-like biomimetic NPs were also shown to simultaneously absorb PAMPs and proinflammatory cytokines, which implies a more potent effect for sepsis management (Thamphiwatana et al., 2017; Wu et al., 2020). Other leukocyte-based biomimetic NPs, nanocarriers, and nanovesicles were demonstrated to enhance inflammation targeting in bacterial sepsis (Zinger et al., 2021; Cao et al., 2022) or otherwise enable anti-inflammation and targeted antiviral treatment in sepsis-associated diseases such as COVID-19 (Tan et al., 2021). Of note, macrophage-membrane-coated polymer NPs (i.e., leukosomes) were shown in a recent work to enhance the anti-inflammatory effect of dexamethasone in a murine endotoxemia model, along with their inherent anti-inflammatory activity due to the contact with macrophages, which skews them into immunosuppressive phenotypes (Molinaro et al., 2020). This further proves the anti-inflammatory efficacy of biomimetic NPs when serving as a potent solution for sepsis treatment.

#### Reversing immunosuppression

Given that sepsis in increasingly recognized as a persistent immunosuppressive condition that threatens patients’ lives at the late stage, the immune-activating activity of membrane-coated biomimetic NPs has also been extensively investigated when selectively utilizing the immunostimulatory functions of coated cell membranes, such as crosstalk with immune cells, remodeling the microenvironment, and the ability of targeted homing. Resident cell membranes and activated cell membranes from macrophages, T cells, or other types of hybrid membranes have been used to coat naked NPs or NPs complexed with other immunomodulatory agents (Parodi et al., 2013; Zeng et al., 2022). For example, a recent study demonstrated the fabrication of a metal-organic framework encapsulated with a plasmid expressing antimicrobial gene LL37 and coated with macrophage membranes. This type of biomimetic NP boosted the antimicrobial response and conferred protection in septic mice bearing immunodeficiency (Cao et al., 2022). Other researchers described the coating of viral mimicking molecules, which enhanced cross-presentation to ameliorate infectious diseases (Molino et al., 2013). Furthermore, NPs were also encapsulated with membrane-mimicking dendrimers that may promote cytokine release (Jatczak-Pawlik et al., 2021) and the uptake of live *Pseudomonas aeruginosa* (Kostina et al., 2019) or preserve the function of immune cells (Kim et al., 2021), which are key indicators reflecting the reversal of immunosuppression in sepsis. Despite these findings, the efficacy of biomimetic NPs has only been tentatively examined in sepsis-associated immunosuppression, warranting more comprehensive investigations in the future.

#### Targeted delivery of immunomodulatory agents

**FIGURE 4:**
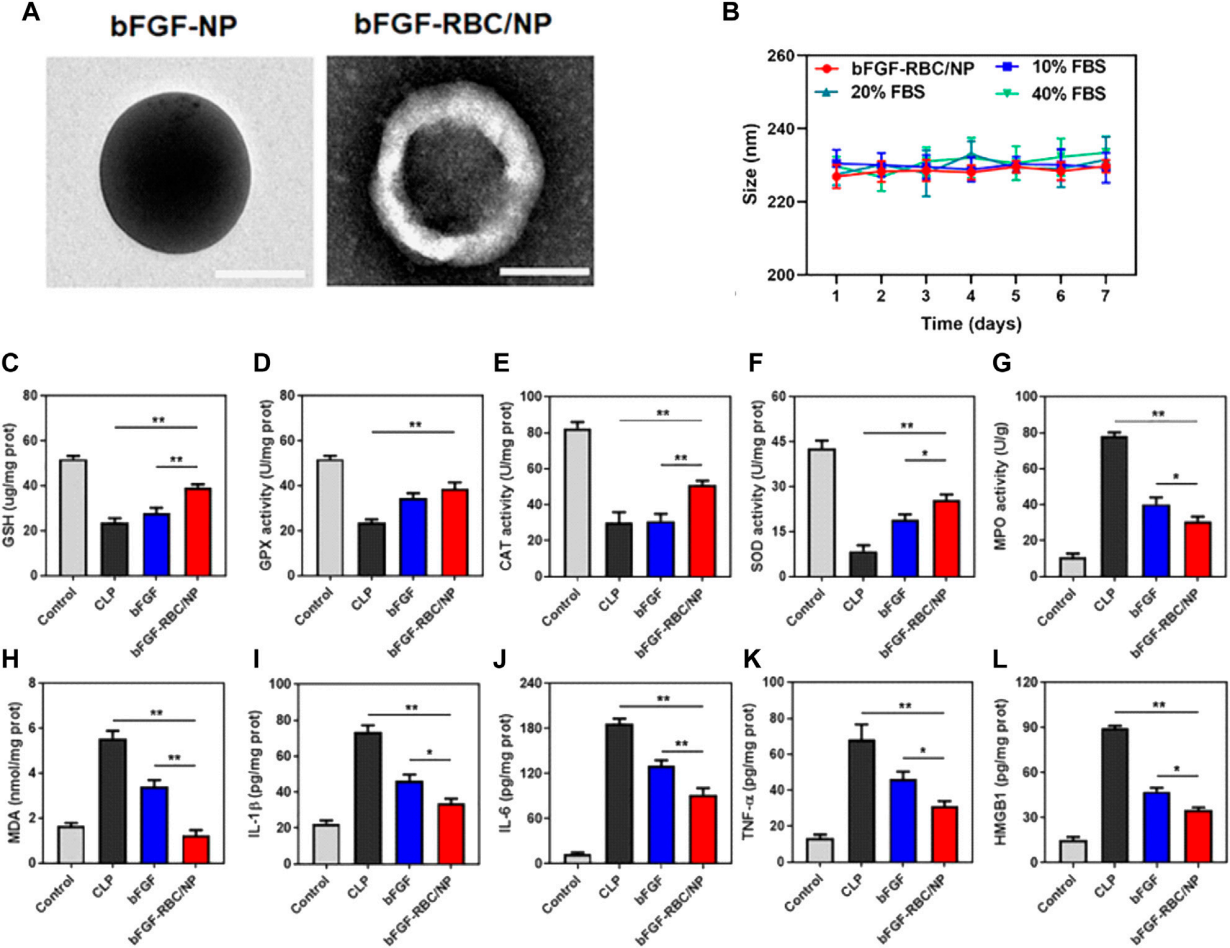
The *in vivo* anti-oxidant and anti-inflammatory effect of RBC membrane coated PLGA NPs carrying basic fibroblast growth factor (bFGF-RBC/NPs) in a murine sepsis model. **(A–B)** TEM and the average hydrodynamic diameters of bFGF-NP and bFGF-RBC/NP (199.7 nm and 226.47 nm respectively). **(C)** Schematic of the injection of bFGF or bFGF-RBC/NP (100 μg/kg) in a murine sepsis model established by cecal ligation and puncture (CLP). **(D–M)** The levels of **(D)** GSH, **(E)** GPX, **(F)** CAT, **(G)** SOD, **(H)** MPO, **(I)** MDA and the contents of **(J)** IL-1β, **(K)** IL-6, **(L)** TNF-α, and **(M)** HMGB1 in heart tissues of CLP mice. Adapted and reproduced with permission from Li et al. (2022b). Copyright^©^ 2022. The authors. Publishing services by Frontiers Media S.A, 2022.

The emergence of NP-coating strategies using biological membranes, accompanied by the inherent advantages of NPs in targeted drug delivery, has prompted investigations of biomimetic delivery with NPs carrying immunomodulatory agents (Balmert and Little, 2012). In contrast to the abovementioned direct immunomodulation by natural or modified membrane-coated biomimetic NPs, this strategy mainly utilizes carrier NPs for the controlled release and targeted delivery of antigens, antibiotics, and anti-inflammatory agents for anti-infectious and anti-inflammatory purposes. In this regard, stability, tolerance, and targeting are priorities when introducing certain types of biological membranes. For example, Lu’s group demonstrated the delivery of an anti-inflammatory and antioxidant agent named the basic fibroblast growth factor *via* an RBC-membrane-coated PLGA NP system (bFGF-RBC/NPs), which showed promise in treating myocardial injury in a murine sepsis model established by CLP (Li et al., 2022). The bFGF-RBC/NPs were also demonstrated to exert the *in vivo* anti-oxidant and anti-inflammatory effect in heart tissues of CLP mice (Figure 4). Moreover, Park et al. (2021) reported their recent work of coating polymeric NP cores with a genetically modified C1498 cell line expressing very late antigen-4 (VLA-4), which established a biomimetic NP carrier for the targeted delivery of dexamethasone to combat lung tissue inflammation *via* the interaction between VLA-4 and vascular cell adhesion molecule-1 (VCAM-1) expressed on inflamed pulmonary endothelial cells. Their findings highlight the potential use of modified membrane-coated NPs to improve the selectivity of delivering anti-inflammatory agents to inflamed sites. An earlier study reported an alternative solution of introducing ghost nanovesicles derived from human U937 monocytes, which similarly delivered the anti-inflammatory dexamethasone to treat bacterial sepsis (Go et al., 2019).

## Conclusion

The past decades have witnessed major breakthroughs in biomedical nanotechnologies and their potential application as tools for controllable immunomodulation in various diseases including sepsis (Fang and Zhang, 2016; Riley et al., 2019; Lasola et al., 2020). A variety of lipid based, polymer-based and inorganic NPs have been approved by FDA for clinical use, including the anti-fungal agent of amphotericin B liposome formulations that treats fungal infection and fungal sepsis (Mitchell et al., 2021). Non-etheless, the applications of NPs have been significantly impeded due to their inherent nature as foreign bodies (Parodi et al., 2020). However, the technique of membrane-coating has demonstrated remarkable enhancements in NP tolerance and stability. These biomimetic NPs have also demonstrated improved performance for immunomodulatory purposes, including anti-infection, inflammation control, reversing of immunosuppression, and targeted delivery with immunomodulatory agents, which strongly indicates their potential application in treating sepsis (Wang et al., 2020). Although most of these investigations are still in their early stages while no marked biomimetic NPs are currently available, biomimetic NPs have already addressed some of the limitations of both conventional therapeutic agents (e.g., poor bioavailability and lack of specific targeting) and naked NPs (e.g., short retention and immunogenicity). In this regard, biomimetic nanomedicine will undoubtedly aid the development of novel sepsis therapeutics.

Despite the encouraging results described above, there is still a long way to go before their actual application in treating sepsis. The first unresolved issue is the uniformity of the particles and the integrity of the coating membranes, thereby significantly affecting the efficacy and safety of the biomimetic NPs due to these inadequacy of quality (Li et al., 2020). Second, the proof of concept for the reproducibility and long-term stability of biomimetic NPs have not been verified, which also greatly affect their translational values into clinical use (Ismail et al., 2022). Third, most of the fabrication process of biomimetic membranes onto NPs was performed in the experimental settings whereas scaling-up techniques needed to be further developed to meet the requirement of large-scale clinical use. Fourth, the biosafety of the biomaterials employed for the design of such bioinspired nanocarriers should be confirmed. The above-mentioned promising findings were exclusively obtained in laboratory in which the sources of membrane or membrane components and the long-term stability and safety of the particles used for therapeutic purposes were not taken into consideration (Papafilippou et al., 2021). Actually, the side effect or off-target effect for the entire particles or the degraded core- and shell-structures as well have not been comprehensively examined. These unanswered questions must be answered before the clinical translation into sepsis therapeutics. On the other hand, challenge may also come from sepsis itself. Standard preclinical animal models are still lacking, which cannot precisely recapitulate the pathophysiology of human sepsis, including infection, inflammation, immunosuppression and organ dysfunction. In this regard, almost all the therapeutic agents which were effective in animal models of sepsis have not proved their efficacy in clinical trials. Consequently, more evidences are needed to prove the advantages of biomimetic NPs in animal models which can be replicated in human patients. Furthermore, sepsis has been unprecedently regarded as a heterogeneous disease, with dynamic changes in inflammation and immune status. Therefore, future efforts are needed to improve the NP coating strategy, to fully evaluate their pharmaceutical properties and to develop diversified biomimetic NPs that may meet the demands of precise therapy for sepsis, rather than simply targeting a specific aspect in the course of sepsis.
